# Hazara orthonairovirus nucleoprotein facilitates viral cell-to-cell spread by modulating tight junction protein, claudin-1

**DOI:** 10.3389/fmicb.2023.1192956

**Published:** 2023-05-23

**Authors:** Keisuke Ohta, Naoki Saka, Masayoshi Fukasawa, Machiko Nishio

**Affiliations:** ^1^Department of Microbiology, School of Medicine, Wakayama Medical University, Wakayama, Japan; ^2^Department of Biochemistry and Cell Biology, National Institute of Infectious Diseases, Tokyo, Japan

**Keywords:** Hazara orthonairovirus, Crimean-Congo hemorrhagic fever virus, nucleoprotein, tight junction, claudin-1

## Abstract

**Background:**

Tight junctions act as a barrier that prevents invasion of pathogens through epithelial cells. This study aims to elucidate the correlation between tight junctions and nairoviruses using Hazara orthonairovirus (HAZV) as a surrogate model for Crimean-Congo hemorrhagic fever virus.

**Methods:**

mRNA, total protein, and cell surface protein levels of tight junction proteins were examined by quantitative real-time reverse transcription polymerase chain reaction, immunoblot and flow cytometry, respectively. HAZV growth was measured by plaque assay. Immunofluorescence assay was used to examine viral cell-to-cell spread. The interaction between HAZV nucleoprotein and claudin-1 was analyzed by immunoprecipitation.

**Results:**

HAZV infection induced mRNA of several tight junction proteins, especially claudin-1. HAZV infection also induced cell surface expression of claudin-1 protein. Claudin-1 overexpression inhibited the growth of HAZV by blocking its cell-to-cell spread. In contrast, HAZV nucleoprotein completely inhibited HAZV-induced cell surface expression of claudin-1, and this inhibition required interaction between HAZV nucleoprotein and claudin-1.

**Conclusion:**

HAZV nucleoprotein was shown to bind to claudin-1 to negatively regulate its cell surface expression, and so can promote cell-to-cell spread of HAZV. This is the first presentation of a possible mechanism behind how nairoviruses counteract tight junction barrier function.

## 1. Introduction

Tight junctions act as a barrier that prevents invasion of pathogens through epithelial cells. The correlation between nairoviruses and tight junctions remains unclear. The family *Nairoviridae*, belonging to the order *Bunyavirales*, includes Crimean-Congo hemorrhagic fever virus (CCHFV).^1^ CCHFV is a cause of severe hemorrhagic fever with a high rate of lethality, and must be handled in a biosafety level (BSL) 4 facility. Hazara orthonairovirus (HAZV) belongs to the same genus as CCHFV (the *Orthonairovirus* genus), but is non-pathogenic to humans and can be handled in a BSL 2 facility. Here, we investigate the effects of tight junctions on the growth of nairoviruses using HAZV as a surrogate model for CCHFV.

HAZV infection, which is similar to CCHFV infection, is lethal to adult type I interferon receptor-knockout mice (Bereczky et al., 2010; Dowall et al., 2012), although these viruses are different BSL pathogens. Moreover, both HAZV and CCHFV have been shown to have high pathogenicity to embryonated chicken eggs (Xia et al., 2013; Matsumoto et al., 2018). HAZV is therefore considered to be a good surrogate model for CCHFV. CCHFV and HAZV are enveloped viruses and their genomes consist of three segments of single-stranded RNA of a negative polarity: S (small), M (medium), and L (large) genes (Elliott and Schmaljohn, 2013). S, M, and L genes encode nucleoprotein (N), glycoprotein (Gn and Gc), and RNA-dependent RNA polymerase (RdRp), respectively.

HAZV N protein consists of two head domains (aa 1–186 and aa 297–485) and one stalk domain (aa 187–296), which is similar to CCHFV N protein (Surtees et al., 2015; Wang et al., 2015). HAZV and CCHFV N proteins have the ability to inhibit apoptosis (Karlberg et al., 2015; Matsumoto et al., 2019). CCHFV N protein exhibits DNA-specific endonuclease activity (Guo et al., 2012). HAZV N protein is also predicted to have this activity because the sites are conserved in HAZV N protein based on its amino acid sequences. We recently reported that HAZV N protein suppresses type I IFN production (Ohta et al., 2022).

Tight junctions act as a barrier between epithelial cells that prevents solutes including pathogens, and they have important roles in compartmentalization between apical and basolateral membrane domains in epithelial cells (Schneeberger and Lynch, 2004). They contain several integral membrane proteins including occludin, junctional adhesion molecules, and claudins (CLDNs) (Furuse et al., 1993, 1998; Martìn-Padura et al., 1998). Zonula occludens (ZO), including ZO-1 and ZO-2, are cytosolic tight junction-related proteins, and are required for the assembly of membrane tight junction proteins, such as CLDNs (Fanning and Anderson, 2009). CLDNs are composed of four transmembrane domains, two extracellular loops, and short cytoplasmic N-and C-terminal domains (Schneeberger and Lynch, 2004). To avoid invasion of pathogens, proinflammatory cytokines induce tight junction proteins; e.g., tumor necrosis factor-α (TNF-α) induces CLDN1 protein, while CLDN1, CLDN2, and occludin are induced by interleukin-1β (IL-1β) (Yamamoto et al., 2004; Al-Sadi and Ma, 2007; Kondo et al., 2008; Fujita et al., 2011).

CLDN1 reportedly inhibits growth of several viruses including herpes simplex virus type 1 (HSV-1) (De Benedetto et al., 2011), human parainfluenza virus type 2 (hPIV-2) (Yumine et al., 2019), and the classical swine fever virus (CSFV) (Wang et al., 2021). In contrast, CLDN1 has been used as co-receptor of hepatitis C virus (HCV), and was shown to be involved in both cell-free and cell-to-cell transmission (Evans et al., 2007; Brimacombe et al., 2011). HCV also utilizes CLDN6 and CLDN9 (Meertens et al., 2008). CLDN1 was reported to be an entry factor of dengue virus (Gao et al., 2010; Che et al., 2013). Occludin is a co-receptor of porcine sapovirus, while this virus disrupts tight junctions for their efficient entry through the basolateral surface of a cell (Alfajaro et al., 2019). The relationship between CCHFV infection and tight junctions has been investigated; CCHFV infection caused neither tight junction disruption, nor altered localization of the tight junction proteins including occludin and ZO-1 (Connolly-Andersen et al., 2007). However, the effects of CLDNs on CCHFV infection have not yet been investigated, and there is currently insufficient direct evidence to prove whether tight junctions affect the growth of CCHFV.

We attempted to identify tight junction proteins that are involved in the growth of HAZV. We present the first report of a possible mechanism behind how nairoviruses counteract host tight junction for their effective growth.

## 2. Materials and methods

### 2.1. Cells and virus

HeLa cells, A549 cells, Madin–Darby canine kidney (MDCK) cells, COS cells, and SW13 cells were grown in Dulbecco’s modified Eagle’s minimal essential medium (DMEM) containing 5% fetal calf serum (FCS). CLDN1 knockdown MDCK (MDCK/CLDN1 KO) and its control (MDCK/ctrl KO) cell lines were previously described (Yumine et al., 2019). All cells were maintained in a humidified incubator at 37̊C with 5% CO_2_. HAZV strain JC280 was used in this study (Begum et al., 1970).

### 2.2. Antibodies

Monoclonal antibodies (MAbs) against HAZV N protein (911–1, used for immunoblot, and 3102-1, used for immunofluorescence assay) were previously described (Matsumoto et al., 2019; Ohta et al., 2022). Anti-actin mAb was purchased from Wako (Osaka, Japan). Anti-CLDN1 polyclonal antibodies (pAbs) used for immunoblot and for immunoprecipitation were purchased from Abcam (Cambridge, United Kingdom) and Proteintech Group (Chicago, IL, United States), respectively. Anti-CLDN1 mAb (7A5) used for flow cytometry was previously described (Fukasawa et al., 2015). Anti-CLDN7 pAb was purchased from Boster Biotechnology (Wuhan, China). Anti-FLAG mAb was obtained from Sigma (St. Louis, MO, United States).

### 2.3. Plasmids

pcDNA3.1 vector (Invitrogen, Carlsbad, CA, United States) carrying HAZV N cDNA was previously described (Matsumoto et al., 2019). Construction of N mutants was performed by standard polymerase chain reaction (PCR) mutagenesis methods. HAZV N cDNA was also cloned into the episomal Epstein–Barr virus-based expression plasmid, pEBS-PL (Bontron et al., 1997). pcDNA3.1 vector carrying CLDN1 cDNA was previously described (Yumine et al., 2019). cDNA of CLDN7 was obtained from total RNA of HeLa cells by reverse transcription polymerase chain reaction (RT-PCR) as previously described (Ohta et al., 2018). CLDN mutants were constructed by a standard PCR mutagenesis method. cDNAs of CLDN1, CLDN7 and their mutants with 3xFLAG tag at their N-termini were cloned into the pCI neo expression vector (Promega, Madison, WI). These constructs were all confirmed by DNA sequencing.

### 2.4. Establishment of HeLa cell line constitutively expressing CLDN1 or N protein

HeLa cells were transfected with pcDNA3.1 carrying CLDN1 or pEBS-PL carrying N cDNA using XtremeGENE HP. Stable transfectants were selected with 100 μg/mL hygromycin (Invitrogen). Expression level of CLDN1 or N protein in each clone was checked by immunoblot, and clones showing high expression levels were used as HeLa cell line constitutively expressing CLDN1 (HeLa/CLDN1) or N protein (HeLa/N).

### 2.5. Quantitative real-time RT-PCR (RT-qPCR)

Total RNAs were isolated from virus-infected cells using Isogen II (Nippon Gene, Tokyo, Japan) according to the manufacturer’s instructions. cDNA synthesis was carried out using a PrimeScript RT reagent kit (Takara, Kyoto, Japan) with oligo-dT primer. cDNAs were subjected to RT-qPCR using Brilliant III Ultra-Fast SYBR Green Master Mix (Agilent Technologies, Santa Clara, CA, United States). The primers used for RT-qPCR are listed in Table 1. Glyceraldehyde-3-phosphate dehydrogenase (GAPDH) was used as an internal control. To amplify the HAZV genome, reverse transcription was performed using the specific primers listed in Table 2. The primers used for RT-qPCR are listed in Table 1. A standard curve was generated from the dilutions with known copy numbers, and the RNA per well of samples was quantified based on this standard curve. To investigate the efficiency of viral entry and genome replication, virus-infected cells were collected at 0.5 and 12 hpi (at early stage of infection), respectively.

**Table 1:** List of primers used for RT-qPCR.

Target gene	Primer sequences (5′–3′)	
	Forward primers	Reverse primers
Human CLDN1	CAGGCTCTCTTCACTGGCTG	TCACACGTAGTCTTTCCCGC
Canine CLDN1	GCTGTCATTGGGGGAGTGAT	CCAGTGAAGAGAGCCTGACC
Human CLDN3	GGGACTTCTACAACCCCGTG	CCGTGTACTTCTTCTCGCGT
Canine CLDN3	CTTCTACAACCCGCTGGTGC	GATCTTGGTGGGCGCGTA
Human CLDN4	ACTTCTACAATCCGCTGGTGG	GCGGAGTAAGGCTTGTCTGT
Canine CLDN4	GTGGCAAGTGCACCAACTG	ACCAGCGGGTTGTAGAAGTC
Human CLDN7	AGTATGAGTTTGGCCCTGCC	ACCCAGCCTTGCTCTCATTC
Canine CLDN7	CGAGTTTGGTCCTGCCATCT	CCTTGGCAGAGTTGGGCTTA
Human ZO-1	GGGACAACAGCATCCTTCCA	ATCACAGTGTGGTAAGCGCA
Canine ZO-1	AAGGTCTGCCGAGACAACAG	CAGGAGTCATGGACGCACAG
Human ZO-2	TGAAGACACGGACGGTGAAG	GGGCTGGGTTTCCTTATGCT
Canine ZO-2	CCTGTCCCAGCACACAAGTT	ATAGCCACGCTTCGAGTGTT
Human occludin	CAGGCCTCTTGAAAGTCCACC	AGGCTGGCTGAGAGAGCATT
Canine occludin	ATAAATCAACACCGGTCCCCG	GGCCTTCCCGTTCTTCCTTT
Human GAPDH	GAAGGTCGGAGTCAACGGATTT	ATCTTGAGGCTGTTGTCATACTTCT
Canine GAPDH	GTCATCCCTGAGCTGAACGG	TCCGATGCCTGCTTCACTAC
HAZV S genome	GATTGCGGCTAGGTTCACTG	AAAGATATCGTTGCCGCACAG
HAZV M genome	GACCATAGCCAGCTAGAGCA	CTCAAAGATATCGTGGCGGC
HAZV L genome	TGTGTCTCCAAACCCACAACT	CCCACACCCCAATTTTAATCTTCT

**Table 2:** List of primers used for RT.

Target gene	Primer sequences (5′–3′)
HAZV S genome	GCCACCAACATCAACATCAT
HAZV M genome	ACTCTATAGCTTCGCCCGC
HAZV L genome	CGGTGTGGAGTGTTGCTGA

### 2.6. Immunoblot and immunoprecipitation analysis

Cells were harvested, sonicated for 30 s three times in lysis buffer containing 50 mM Tris–HCl (pH7.4), 150 mM NaCl, 0.6% NP-40, and proteinase inhibitor cocktail (cOmplete, Roche, Basel, Switzerland), and then centrifuged. Cell lysates were separated by sodium dodecyl sulfate-polyacrylamide gel electrophoresis, transferred to a nitrocellulose membrane, and analyzed by western blot (WB) technique, as previously described (Ohta et al., 2016). For immunoprecipitation, the supernatants were incubated with nProtein A Sepharose 4 Fast Flow (GE healthcare Bio-Sciences, Piscataway, NJ, United States) preincubated with the appropriate Abs. Precipitated proteins were analyzed by WB.

### 2.7. Flow cytometry

HeLa cells were harvested with DMEM containing 0.53 mM EDTA and 0.05% trypsin and washed with phosphate-buffered saline (PBS) containing 2% FCS and 0.1% sodium azide. Cells were then reacted with anti-CLDN1 mAb or anti-CLDN7 pAb for 1 h followed by Alexa Fluor 488 goat anti-mouse IgG (Invitrogen) for 1 h. After being fixed with 1% paraformaldehyde, cells were analyzed with a FACSCalibur cytometer (BD Biosciences, San Jose, CA, United States).

### 2.8. Plaque assay

SW13 cells grown in 12-well plates were infected with HAZV diluted serially 10-fold in DMEM without FCS and then cultured in DMEM containing 2% FCS, 0.5% SeaKem ME agarose, and 0.5% SeaPlaque agarose (FMC Bioproducts, Rockland, ME, United States) for 4–5 days. The cells were then stained with 0.3% amide black.

### 2.9. Immunofluorescence assay

Immunofluorescence assay to examine viral cell-to-cell spread was performed as described previously (Yumine et al., 2019), but with minor modifications. Briefly, HAZV-infected cells were cultured in DMEM containing 2% FCS and 0.5% SeaKem ME agarose. The cells were fixed with 4% paraformaldehyde, and were permeabilized with 0.2% TritonX-100. The cells were then incubated with anti-N mAb, followed by the secondary antibody reaction with AlexaFluor 488 goat anti-mouse IgG (Invitrogen). The cells were mounted with Fluoromount-G (SouthernBiotech, Birmingham, AL, United States) and analyzed by a fluorescence microscope (BZ-X810; Keyence Co., Osaka, Japan).

## 3. Results

### 3.1. HAZV infection induces cell surface expression of CLDN1

**Figure 1:**
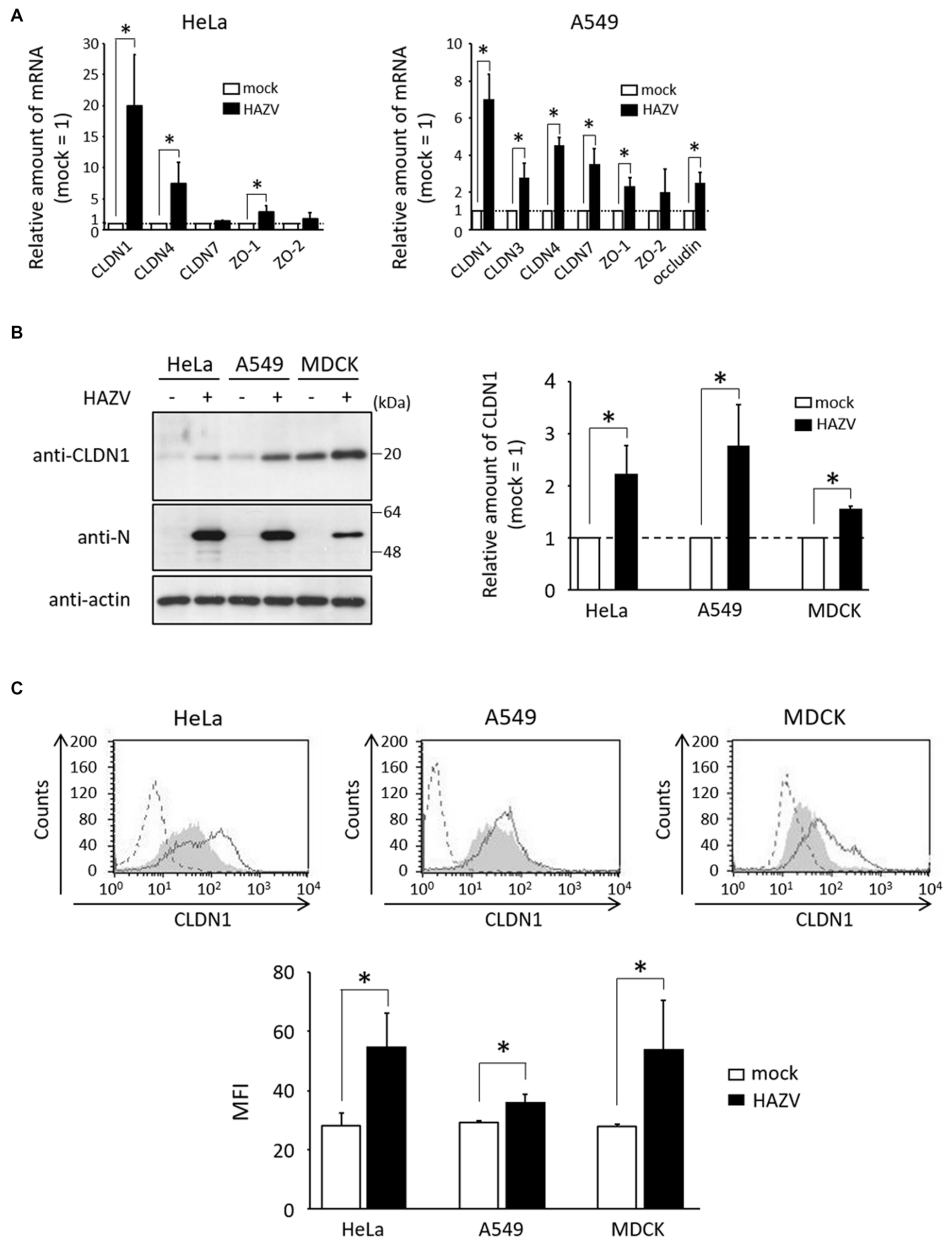
Effects of HAZV infection on tight-junction proteins. **(A)** HeLa cells and A549 cells were infected with HAZV at the MOIs of 1.0 and 0.1, respectively. After 48 h, expression of the indicated mRNAs was measured by RT-qPCR. Data are the means from three independent experiments, and are shown as the relative value (mock = 1). Error bars indicate standard deviations. *p*-values were calculated by the Student’s *t*-test. *p < 0.05, compared with values of mock cells. **(B)** HAZV was inoculated into HeLa cells (MOI of 1.0), A549 cells (MOI of 0.1), and MDCK cells (MOI of 1.0) for 48 h, and lysates were subjected to immunoblot using the indicated Abs. Actin was used as a loading control. Bars show the quantitative densitometry of CLDN1 protein performed using ImageJ software (http://rsb.info.nih.gov/ij). Data are the means from three independent experiments, and are shown as the relative value (mock = 1). *p*-values were calculated by the Student’s *t*-test. *p < 0.05, compared with values of mock cells. Error bars indicate standard deviations. **(C)** HeLa cells, A549 cells, and MDCK cells were infected with HAZV as described in **(B)**, and were subjected to flow cytometry using anti-CLDN1 Ab. Mock cells and HAZV infected cells are indicated as gray shading and black lines, respectively. Cells not treated with anti-CLDN1 Ab are indicated by a dashed line. Bars show the geometric mean fluorescence intensity (MFI) of CLDN1. Data are the means from three independent experiments, and are presented as the relative MFI (mock = 1). *p*-values were calculated by the Student’s *t*-test. *p < 0.05, compared with values of mock cells. Error bars indicate standard deviations.

In HeLa cells, the mRNA of CLDN1 was especially sensitive to the HAZV infection, with approximately 20-fold increase (Figure 1A). HAZV infection also up-regulated CLDN4 and ZO-1 mRNAs, but did not affect CLDN7 and ZO-2 mRNAs (Figure 1A). mRNAs of CLDN3 and occludin were not detected in either mock- or HAZV-infected HeLa cells (data not shown). Similar experiments were performed using A549 cells. The increase of CLDN1 mRNA by HAZV infection was the most noticeable also in A549 cells (Figure 1A). In MDCK cells with a high endogenous expression of CLDN1 protein (Yumine et al., 2019), HAZV infection did not cause a remarkable increase in CLDN1 mRNA (data not shown).

CLDN1 was the most up-regulated gene by HAZV infection, so we next investigated whether HAZV infection induces CLDN1 protein expression. HAZV infection resulted in the increase of CLDN1 protein level (approximately twofold) (Figure 1B). CLDN1 is a membrane protein, so we also quantified cell surface CLDN1 by flow cytometry. The histogram of cells infected with HAZV showed a clear rightward shift, indicating an increase of cell surface CLDN1 expression (Figure 1C). In addition to HeLa cells, similar results were obtained in A549 cells and MDCK cells (Figures 1B,C).

### 3.2. CLDN1 inhibits the growth of HAZV

**Figure 2:**
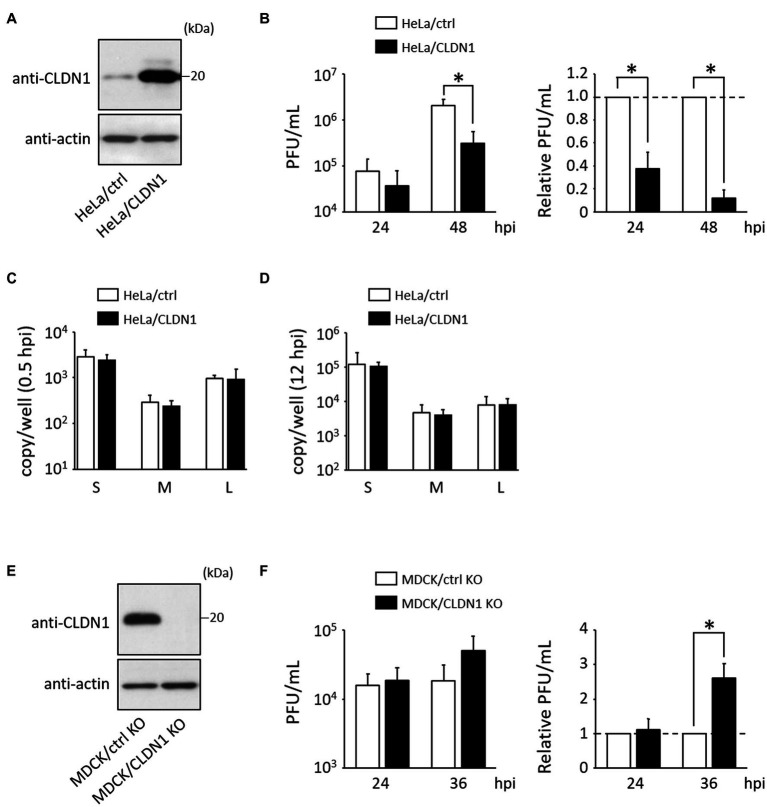
Effects of CLDN1 on the growth of HAZV. **(A,E)** Lysates of the indicated cell lines were subjected to immunoblot using anti-CLDN1 Ab. Actin was used as a loading control. **(B)** HeLa/ctrl and HeLa/CLDN1 were infected with HAZV at an MOI of 0.1 for the indicated hours, and the virus titers were determined by plaque assay. PFU/mL values are shown as the means from three independent experiments. Relative PFU/mL values are also shown (HeLa/ctrl = 1). *p*-values were calculated by the Student’s *t*-test. *p < 0.05 compared with values of HeLa/ctrl. Error bars indicate standard deviations. **(C,D)** HeLa/ctrl and HeLa/CLDN1 were incubated with HAZV at an MOI of 0.1 for 0.5 h **(C)** or for 12 h **(D)**, and total RNA was extracted using Isogen II. Copy numbers of HAZV genome were measured by RT-qPCR. Data are the means from three independent experiments. Error bars indicate standard deviations. **(F)** MDCK/ctrl KO and MDCK/CLDN1 KO were infected with HAZV at an MOI of 0.1 for the indicated hours, and plaque assay was performed as described in **(B)**. Relative PFU/mL values are also shown (MDCK/ctrl KO = 1).

To examine whether induction of CLDN1 affects HAZV growth, a HeLa cell line constitutively overexpressing CLDN1 (HeLa/CLDN1) was generated (Figure 2A). Virus production in HeLa/CLDN1 was approximately 3- and 10-fold lower than that in the control cell line (HeLa/ctrl) at 24 and 48 h post-infection (hpi), respectively (Figure 2B). Next, we assessed whether viral entry and genome replication steps are affected by CLDN1 overexpression. The HAZV genomes were quantified by RT-qPCR, as detailed in part 2.5 of the Materials and methods section. The amounts of HAZV genomes in HeLa/CLDN1 were similar to those in HeLa/ctrl at both 0.5 and 12 hpi (Figures 2C,D), indicating that CLDN1 affects neither viral entry nor genome replication. To further confirm the influence of CLDN1 in HAZV growth, MDCK/CLDN1 KO was used (Figure 2E). At 36 hpi, virus production was increased approximately threefold by CLDN1 KO (Figure 2F). CLDN1 KO affected neither viral entry nor genome replication (data not shown). HAZV-induced CLDN1 expression was indicated by these results to decrease the virion production in the supernatants without affecting virus entry or genome replication.

### 3.3. CLDN1 inhibits cell-to-cell spread of HAZV

**Figure 3:**
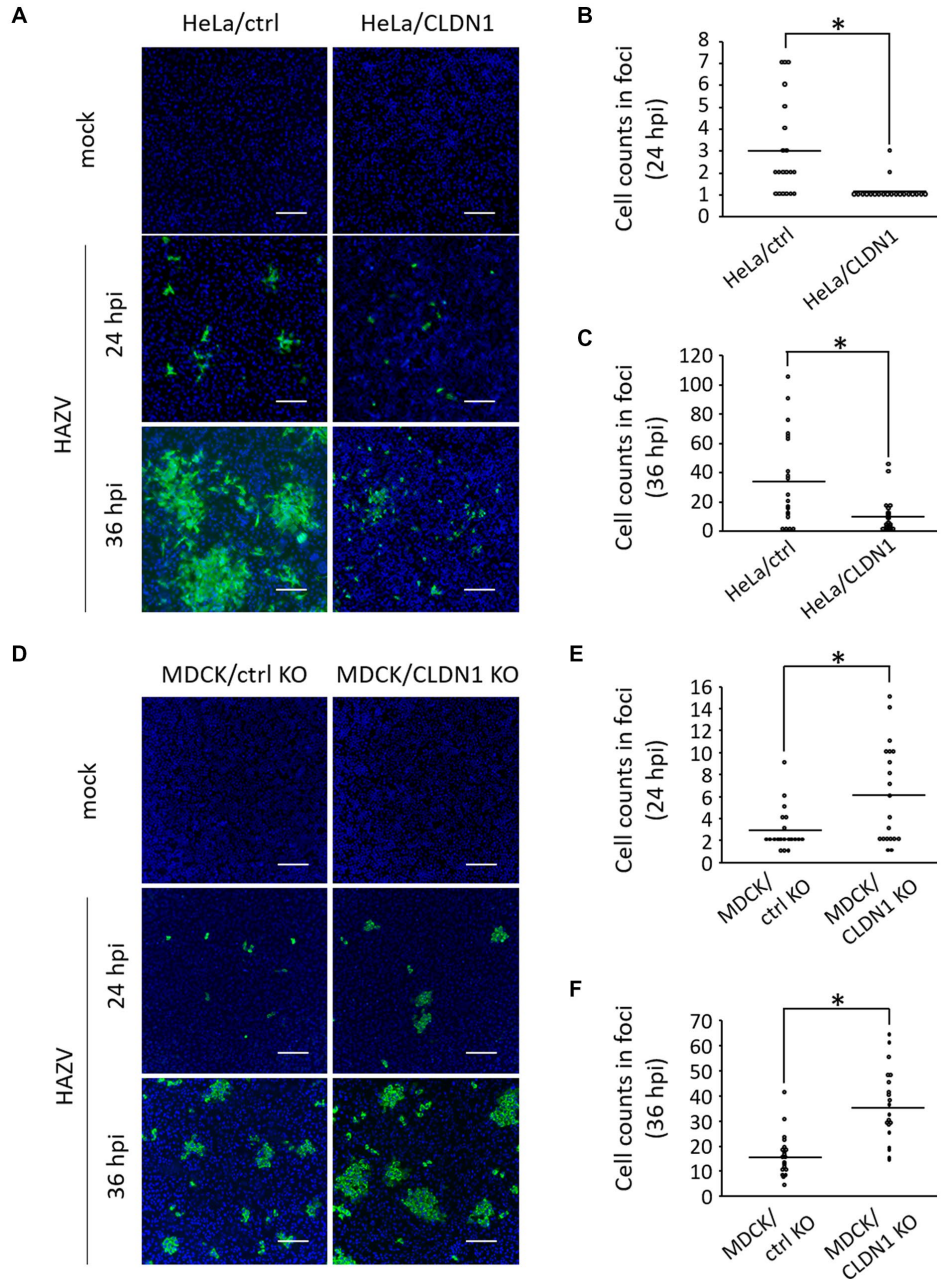
Effects of CLDN1 on the cell-to-cell spread of HAZV. **(A)** HeLa/ctrl and HeLa/CLDN1 were infected with HAZV at an MOI of 0.001 for 24 and 36 h, and were fixed, permeabilized, and stained with anti-N mAb (green). Nuclei were stained with DAPI (blue). Scale bar, 100 μm. **(B,C)** The cell numbers of HAZV infected foci in **(A)** were counted, and the cell counts of twenty foci per each cell line are shown. Results of 24 hpi **(B)** and 36 hpi **(C)** are shown. *p*-values were calculated by the Student’s *t*-test. *p < 0.05 compared with values of HeLa/ctrl. **(D)** MDCK/ctrl KO and MDCK/CLDN1 KO were infected with HAZV, and immunofluorescence assay was performed as described in **(A)**. **(E,F)** Cell counts of HAZV infected foci in **(D)** at 24 hpi **(E)** and 36 hpi **(F)** were performed as described in **(B,C)**. *p < 0.05 compared with values of MDCK/ctrl KO.

We investigated the possibility that CLDN1 inhibits cell-to-cell spread of HAZV by immunofluorescence assay of HAZV-infected HeLa/ctrl and HeLa/CLDN1 cultured in agarose-containing medium. Some of HAZV-infected HeLa/ctrl cells were observed as foci consisting of several cells at 24 hpi, and they widely expanded at 36 hpi (Figure 3A, HeLa/ctrl, Figures 3B,C). In contrast, HAZV-infected HeLa/CLDN1 cells were scattered as single cells at 24 hpi (Figure 3A, HeLa/CLDN1, 24 hpi and Figure 3B). Even at 36 hpi, their spread was modest, and the number of the cells in foci was significantly low relative to HeLa/ctrl (Figure 3A, HeLa/CLDN1, 36 hpi and Figure 3C). Similar experiments were performed using MDCK/CLDN1 KO. CLDN1 KO facilitated cell-to-cell spread of HAZV at both 24 and 36 hpi (Figures 3D–F). CLDN1 was shown by these results to inhibit cell-to-cell spread of HAZV.

### 3.4. HAZV N protein inhibits cell surface expression of CLDN1

**Figure 4:**
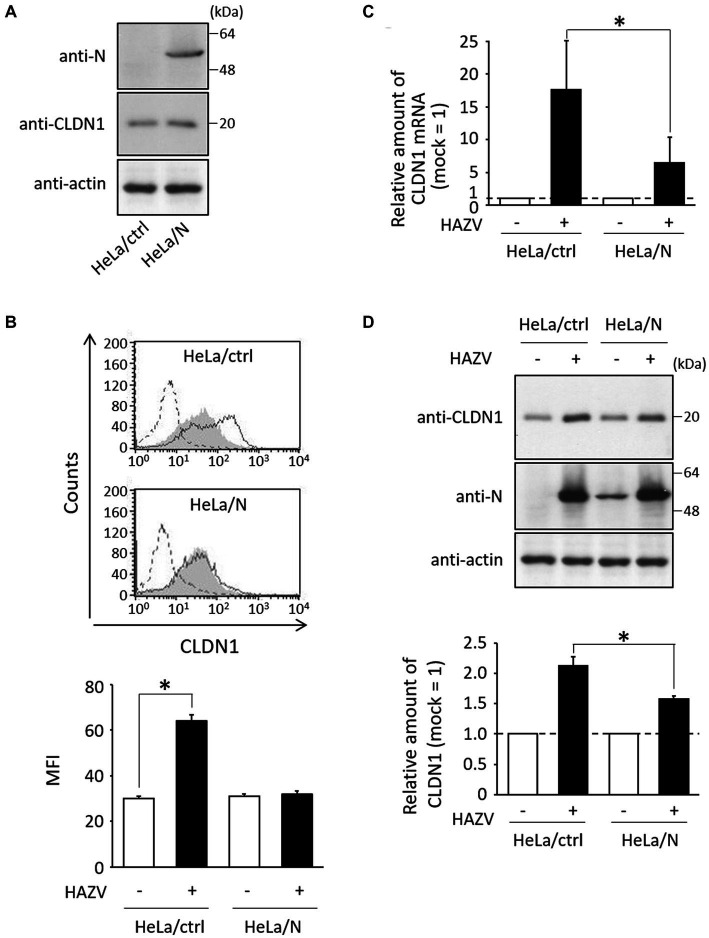
Effects of N protein on HAZV-induced CLDN1. **(A)** Lysates of the indicated cell lines were subjected to immunoblot using the indicated Abs. Actin was used as a loading control. **(B)** The indicated cell lines were infected with HAZV at an MOI of 1 for 48 h, and flow cytometry using anti-CLDN1 Ab was performed as described in Figure 1C. *p < 0.05, compared with values of mock cells. Error bars indicate standard deviations. **(C)** HAZV infection was performed as described in **(B)**, and CLDN1 mRNA was quantified as described in Figure 1A. *p < 0.05, compared with values of HAZV-infected HeLa/ctrl. **(D)** HAZV infection was performed as described in **(B)**, and immunoblot and the quantitative densitometry of CLDN1 protein were performed as described in Figure 1B. *p < 0.05, compared with values of HAZV-infected HeLa/ctrl. Error bars indicate standard deviations.

Next, we assessed virus strategies for dealing with CLDN1. To investigate the effects of N protein, we generated HeLa cell line constitutively expressing N protein (HeLa/N) (Figure 4A). Surprisingly, the histogram of HAZV-infected HeLa/N overlapped with that of mock cells (Figure 4B), indicating that N protein completely suppressed induction of cell surface CLDN1 by HAZV infection. mRNA and total protein expression of CLDN1 by HAZV infection between HeLa/ctrl and HeLa/N was also compared using RT-qPCR and immunoblot, respectively. CLDN1 mRNA in HeLa/N was induced by HAZV infection, but this induction was more modest than that observed in HeLa/ctrl (Figure 4C). Overexpression of N protein also slightly inhibited induction of CLDN1 protein by HAZV infection (Figure 4D). N protein is indicated by these results to strongly suppress cell surface expression of CLDN1 with only a partial inhibition of induction of CLDN1 mRNA and protein.

### 3.5. HAZV N protein binds to CLDN1

**Figure 5:**
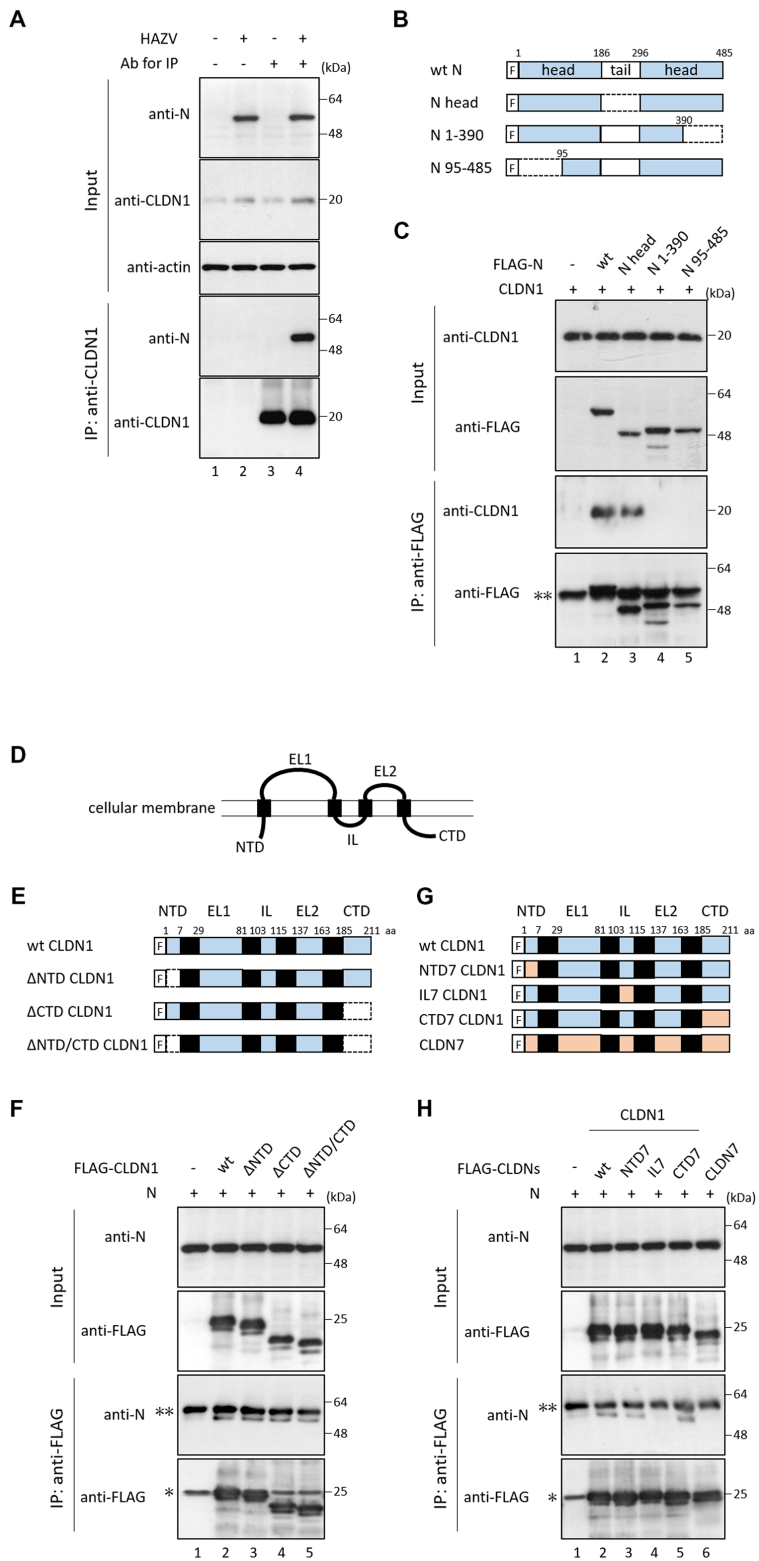
Interaction between CLDN1 and HAZV N protein. **(A)** HeLa cells were infected with HAZV at an MOI of 1.0 for 24 h, and the cell lysates were directly analyzed by immunoblot (input). The cell lysates were immunoprecipitated with or without anti-CLDN1 Ab, followed by immunoblot. Actin was used as a loading control. **(B)** Schematic diagrams of wt N protein and its mutants. “F” indicates FLAG tag. **(C,F,H)** COS cells were transfected with the indicated plasmids. After 48 h, cell lysates were directly analyzed by immunoblot (input). The cell lysates were immunoprecipitated with anti-FLAG mAb, followed by immunoblot. “*” and “**” indicate immunoglobulin light chain and heavy chain, respectively. All experiments were performed at least three times independently. **(D)** Schematic presentation of CLDN structure. CLDNs are composed of four transmembrane domains. A N-terminal intracellular domain (NTD) is followed by extracellular loop 1 (EL1), intracellular loop (IL), EL2, and C-terminal intracellular domain. **(E,G)** Schematic diagrams of wt CLDN1, its mutants, and CLDN7. “F” indicates FLAG tag. Deleted regions are indicated by dotted lines.

We investigated the interaction between N protein and CLDN1 in HAZV-infected HeLa cells by immunoprecipitation. N protein was co-precipitated by anti-CLDN1 Ab in HAZV-infected cells (Figure 5A, lane 4). Next, we determined the regions of N protein important for the binding with CLDN1 using N deletion mutants (Figure 5B). N protein or its deletion mutants were co-expressed with FLAG-tagged CLDN1 in COS cells. A deletion mutant consisting of only two head domains (N head) could bind to CLDN1 (Figure 5C, lane 3), while deletion of either N- or C-terminal region lost the binding activity to CLDN1 (Figure 5C, lanes 4 and 5), suggesting that both of the two head domains are necessary for binding with CLDN1. Next, to determine the regions of CLDN1 important for binding with N protein, we prepared deletion mutants of CLDN1 without N-terminal (ΔNTD), C-terminal intracellular domain (ΔCTD), or both of them (ΔNTD/CTD) (Figures 5D,E). All of these CLDN1 mutants as well as wt CLDN1 bound to N protein (Figure 5F, lanes 2–5). Immunoprecipitation was then performed using CLDN1/7 chimeric mutants (Figure 5G). CLDN7 did not bind to N protein (Figure 5H, lane 6). Substitution of either NTD or CTD of CLDN1 with that of CLDN7 retained binding capacity with N protein (Figure 5H, lanes 3 and 5). In contrast, IL7 CLDN1, the intracellular loop of which was replaced with that of CLDN7, did not bind to N protein (Figure 5H, lane 4). The intracellular loop of CLDN1 is thus indicated to be important for binding with N protein.

### 3.6. N-CLDN1 interaction is necessary for inhibition of cell surface expression of CLDN1 by N protein

**Figure 6:**
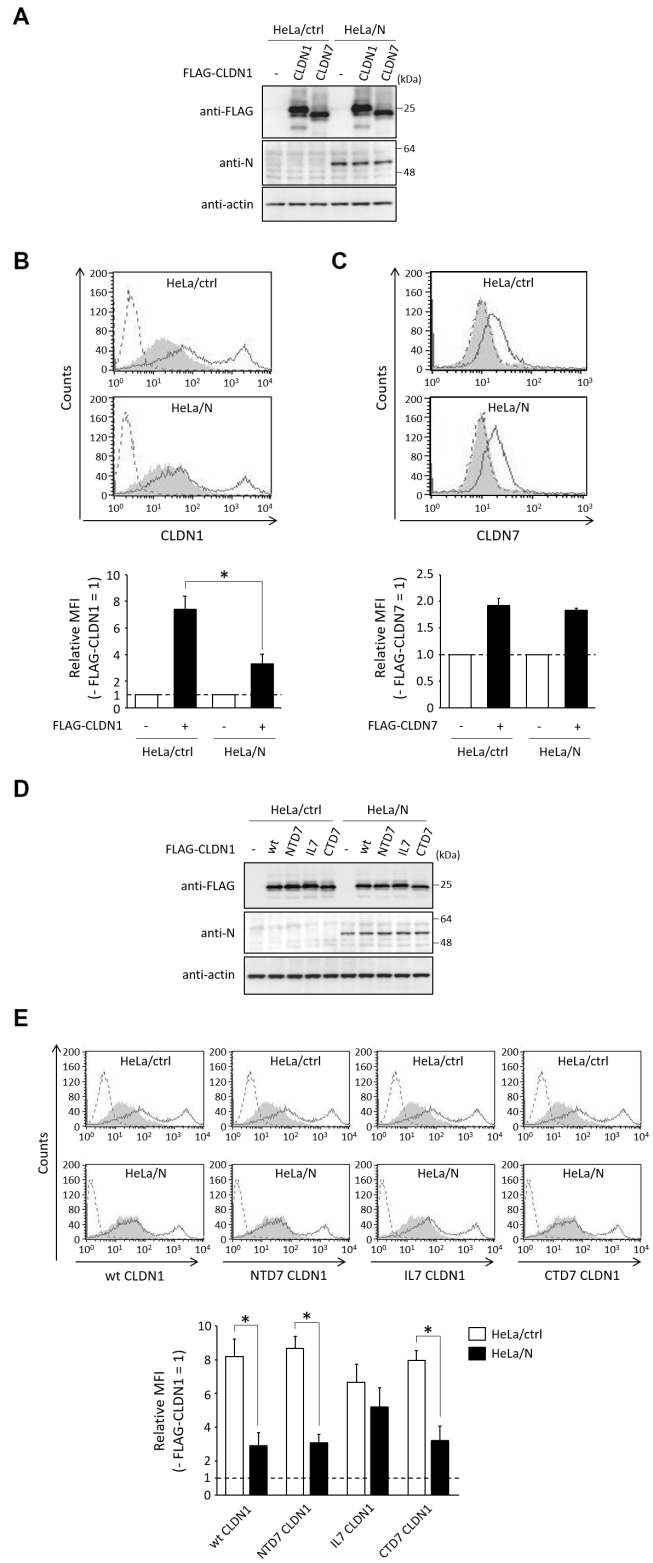
Effects of N protein on the expression of CLDNs. **(A,D)** HeLa/ctrl and HeLa/N were transfected with the indicated plasmids. After 24 h, immunoblot was performed as described in Figure 1B. Actin was used as a loading control. **(B,C,E)** HeLa/ctrl and HeLa/N were transfected as described in **(A)**, and subjected to flow cytometry using anti-CLDN1 **(B,E)** or anti-CLDN7 **(C)**, as described in Figure 1C. Cells transfected with empty vector and pCI neo-FLAG-CLDN1 are indicated as gray shading and black lines, respectively. Cells not treated with anti-CLDN1 or anti-CLDN7 Ab are indicated by a dashed line. Bars show the MFI of transfected CLDNs. Data are represented as relative values (− FLAG-CLDNs = 1). *p < 0.05, compared with values of HeLa/ctrl. Error bars indicate standard deviations.

To investigate whether N protein also inhibits cell surface expression of FLAG-tagged CLDNs, FLAG-CLDN1, or FLAG-CLDN7 was expressed in HeLa/ctrl and HeLa/N. The total and cell surface expression levels of CLDNs between these cell lines were compared using immunoblot and flow cytometry, respectively. Total protein levels of CLDN1 and CLDN7 in HeLa/N were similar to those in HeLa/ctrl (Figure 6A). N protein also inhibited cell surface expression of transfected CLDN1 (Figure 6B), but it did not affect CLDN7 cell surface expression (Figure 6C). We next investigated CLDN1/7 chimeric mutants (Figure 5G). Total protein levels of CLDN1/7 chimeric mutants in HeLa/N were similar to those in HeLa/ctrl (Figure 6D). N protein could similarly inhibit cell surface expression of NTD7 CLDN1 and CTD7 CLDN1, as well as of wt CLDN1 (Figure 6E). In contrast, N protein did not affect cell surface expression of IL7 CLDN1 (Figure 6E). N-CLDN1 interaction is indicated to be necessary for inhibition of cell surface expression of CLDN1 by N protein.

## 4. Discussion

We investigated whether HAZV infection affects CLDN1 expression using HeLa cells, A549 cells, and MDCK cells. HAZV infection in A549 cells was performed at a lower multiplicity of infection (MOI) (MOI of 0.1) than other cells (MOI of 1.0) (Figure 1) because HAZV-infected A549 cells at an MOI of 1.0 showed severe cytopathic effect. HAZV infection caused remarkable increases in CLDN1 mRNA in HeLa cells and in A549 cells (Figure 1A), so we focused on CLDN1. Increase of CLDN1 mRNA and protein in MDCK cells by HAZV infection was modest (Figure 1B and data not shown). MDCK cells could possibly be insensitive to induction of CLDN1 mRNA and protein because they abundantly express CLDN1 protein (Figure 1B). HAZV infection was found to induce cell surface expression of CLDN1 (Figure 1C), resulting in inhibition of HAZV growth (Figures 2B,F). CLDN1 also negatively regulates growth of other viruses including HSV-1 (the family *Herpesviridae*) (De Benedetto et al., 2011), hPIV-2 (the family *Paramyxoviridae*) (Yumine et al., 2019), and CSFV (the family *Flaviviridae*) (Wang et al., 2021). Furthermore, paracellular penetration of human papillomavirus 16 (the family *Papillomaviridae*) was promoted in the presence of Tat and gp120 proteins of human immunodeficiency virus type 1 (HIV-1) that disrupt tight junction proteins including CLDN1 (Tugizov et al., 2013). CLDN1 is suggested by this data to act as an inhibitory factor for a variety of viruses across the family.

We also investigated whether HAZV can counteract the negative effects of CLDN1 on virus growth. HAZV N protein completely inhibited the induction of cell surface expression of CLDN1 by HAZV infection (Figure 4B). CLDN1 is an entry factor for HCV and dengue virus (Evans et al., 2007; Gao et al., 2010; Che et al., 2013). Entry efficiency of HAZV in HeLa/CLDN1 was similar to that in HeLa/ctrl (Figure 2C), indicating that HAZV does not utilize CLDN1 for its entry. CLDN1 was reported to undergo degradation by capsid proteins of West Nile virus and Japanese encephalitis virus through proteasome-mediated pathway (Medigeshi et al., 2009; Agrawal et al., 2013). Infection of CSFV also causes degradation of CLDN1 (Wang et al., 2021). Furthermore, gp120 of HIV-1 degrades other tight junction proteins, ZO-1 and ZO-2 (Nakamuta et al., 2008). However, overexpression of HAZV N protein did not affect CLDN1 protein expression level (Figure 4A), suggesting that HAZV N protein does not induce CLDN1 degradation. CLDN1 is thought to be transported to the cellular membrane through endoplasmic reticulum-Golgi pathway, like normal membrane proteins (Hoevel et al., 2002). Furthermore, CLDN1 reportedly undergoes endocytosis and is recycled back to the cellular membrane (Dukes et al., 2011). N protein might inhibit transport of CLDN1 to the cellular membrane via the interaction with CLDN1 within the cytoplasm. N protein bound to intracellular loop of CLDN1 but not to that of CLDN7 (Figure 5H). The intracellular loop of CLDNs is comprised of 13 amino acid residues (aa 103–115) (Figure 5E), and six amino acid residues among them differ between CLDN1 and CLDN7. These residues are thus predicted to be binding sites for N protein.

Both of the two head domains of HAZV N protein (aa 1–186 and aa 297–485) are necessary for the interaction with CLDN1 (Figure 5C, lanes 3–5). N proteins of HAZV and CCHFV are structurally similar (Surtees et al., 2015; Wang et al., 2015), and the amino acid sequence between the head domains of HAZV N protein and CCHFV N protein is more than 60% identical. CCHFV N protein might therefore also bind to CLDN1 to reduce its cell surface expression.

Our results collectively suggest that cell surface expression of CLDN1 was induced by HAZV infection, which inhibits cell-to-cell spread of HAZV. Moreover, to counteract this, N protein binds to CLDN1 to inhibit its cell surface expression, thus promoting HAZV growth via facilitating its cell-to-cell spread. Unexpectedly, N protein seemed to slightly inhibit cell surface expression of IL7 CLDN1, despite the loss of ability to bind to N protein (Figures 5H, 6E). Palmitoylation of two Cys residues within the intracellular loop of CLDN14 was reported to be important for its efficient localization at the cellular membrane (Van Itallie et al., 2005). These Cys residues are conserved among all CLDNs (Van Itallie et al., 2005), so intracellular loop of other CLDNs also has the potential to contribute to their efficient cell surface expression. Furthermore, unlike CLDN1, CLDN7 is reported to primarily localize at the basolateral surface (Fujita et al., 2006). Substitution of intracellular loop of CLDN1 with that of CLDN7 might therefore result in abnormal trafficking, leading to unexpected inhibition of cell surface expression of IL7 CLDN1 by N protein.

HAZV-induced expression of CLDN1 mRNA in HeLa/N was partially inhibited compared with that of HeLa/ctrl, although it was incomplete (Figure 4C). A moderate inhibition of HAZV-induced CLDN1 protein in HeLa/N (Figure 4D) would result from inhibition of CLDN1 mRNA induction. CLDN1 was reported to be induced by proinflammatory cytokines including TNF-α and IL-1β (Al-Sadi and Ma, 2007; Kondo et al., 2008). We previously reported that HAZV N protein acts as a regulator of host cell activity, such as by inhibition of apoptosis and type I IFN production (Matsumoto et al., 2019; Ohta et al., 2022). N protein likely indirectly suppresses induction of CLDN1 mRNA by blocking induction of proinflammatory cytokines. Induction level of CLDN1 protein by HAZV infection in A549 cells was higher than that in HeLa cells and MDCK cells (Figure 1B). This might be due to the effects of the proinflammatory cytokines, because A549 cells are sensitive to the action of proinflammatory cytokines (Standiford et al., 1990).

HIV-1, West Nile virus, and Japanese encephalitis virus disrupt tight junctions (Nakamuta et al., 2008; Medigeshi et al., 2009; Agrawal et al., 2013). Furthermore, disruption of tight junctions reportedly promoted cell-to-cell spread of HSV-1 (Sufiawati and Tugizov, 2014). Disturbance of tight junctions can cause hemorrhages, probably due to an increased permeability of endothelium (Bazzoni and Dejana, 2004). However, CCHFV infection does not induce disruption of tight junctions (Connolly-Andersen et al., 2007), so the hemorrhages observed in patients with CCHF might be induced by other mechanisms. Nairoviruses seem not to cause drastic change of tight junctions such as its disruption, but to regulate expression of tight junction protein(s). Further study is needed to investigate whether and how these tight junction proteins are involved in nairovirus infection.

## Data availability statement

The original contributions presented in the study are included in the article/supplementary material, further inquiries can be directed to the corresponding author.

## Author contributions

KO and MN contributed to the conception and design of the study. KO and NS performed the experiments. KO wrote the first draft of the manuscript. KO, NS, MF, and MN contributed to the manuscript revision, read, and approved the manuscript. All authors contributed to the article and approved the submitted version.

## Funding

This work was supported by the Grant-in-Aid Scientific Research from the Ministry of Education, Culture, Sports, Science and Technology, Japan (20K07528).

## Conflict of interest

The authors declare that the research was conducted in the absence of any commercial or financial relationships that could be construed as a potential conflict of interest.

## Publisher’s note

All claims expressed in this article are solely those of the authors and do not necessarily represent those of their affiliated organizations, or those of the publisher, the editors and the reviewers. Any product that may be evaluated in this article, or claim that may be made by its manufacturer, is not guaranteed or endorsed by the publisher.

## Footnotes

^1^ http://talk.ictvvvonline.org/taxonomy
